# Cost-effectiveness and public health impact of recombinant zoster vaccine versus no herpes zoster vaccination in selected populations of immunocompromised adults in Canada

**DOI:** 10.1186/s12913-025-12550-x

**Published:** 2025-04-25

**Authors:** Sydney George, Justin Carrico, Katherine A. Hicks, Dessi Loukov, Cheryl Ng, Desmond Curran

**Affiliations:** 1 GSK, Mississauga, ON Canada; 2 RTI-Health Solutions, Research Triangle Park, NC USA; 3 GSK, Singapore, Singapore; 4 GSK, Wavre, Belgium

**Keywords:** Recombinant zoster vaccine, Immunocompromised, Hematopoietic stem-cell transplant, Breast cancer, Renal transplant, Human immunodeficiency virus, Hodgkin lymphoma, Canada

## Abstract

**Background:**

The risk of herpes zoster (HZ) increases with age and in immunocompromised (IC) patients. Recombinant zoster vaccine (RZV) is currently recommended in Canada for people aged ≥ 50 years. The objectives of the current study were to evaluate the cost-effectiveness and public health impact of RZV versus no HZ vaccination in select Canadian IC adult populations.

**Methods:**

The ZOster ecoNomic Analysis ImmunoCompromised (ZONA IC) model followed a base-case cohort of 1600 patients with hematopoietic stem-cell transplant (HSCT) from a starting age of 55 years, who maintained IC status for 5 years, from a societal perspective. Scenario analyses were conducted for patients with breast cancer, renal transplant, human immunodeficiency virus (HIV), and Hodgkin lymphoma. These probabilistic analyses used a life-long time horizon and discount rates of 1.5% for costs and quality-adjusted life-years (QALYs). First-dose coverage was assumed to be 60% and second-dose completion 100%. Deterministic one-way sensitivity analysis for the base case was performed. Costs are reported in 2022 Canadian dollars, with an assumed cost-effectiveness threshold of $50,000 per QALY gained.

**Results:**

In the base-case analysis (HSCT), it was estimated that RZV would prevent medians of 116 HZ and 27 postherpetic neuralgia (PHN) cases, respectively versus no HZ vaccination. Estimated median numbers needed to vaccinate were 8 and 35 to avoid one HZ and one PHN case, respectively. The median incremental cost-effectiveness ratio (ICER) was $22,648 per QALY gained and was most sensitive to assumptions of HZ incidence, direct medical costs for unvaccinated HZ without PHN, and RZV efficacy against PHN. In other IC populations, estimated median ICERs were $24,328 (breast cancer), $27,237 (renal transplant), $67,207 (HIV), and $81,470 (Hodgkin lymphoma).

**Conclusions:**

RZV in Canada improves public health outcomes and is likely cost-effective for several IC conditions.

**Graphical Abstract:**

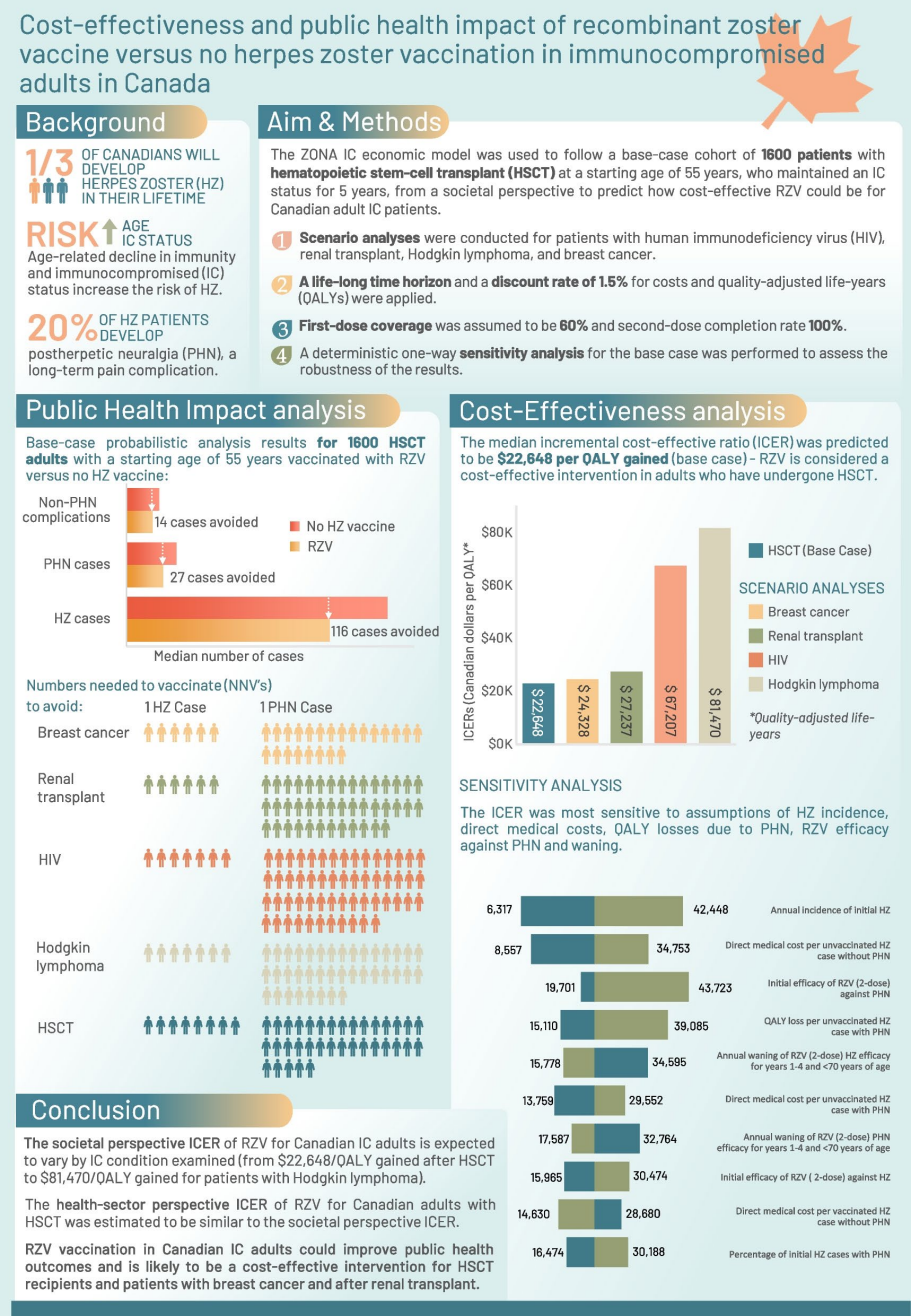

**Supplementary Information:**

The online version contains supplementary material available at 10.1186/s12913-025-12550-x.

## Background

Nearly 1 in 3 Canadians will develop herpes zoster (HZ) in their lifetime [1]. This equates to approximately 130,000 cases each year, including 17,000 cases of postherpetic neuralgia (PHN)– a common and severe complication of HZ– and 20 deaths [2]. The risk of HZ increases with age due to a natural waning of immune function, with annual incidences increasing from around 5 per 1000 people aged 50–54 years to 11 per 1000 people aged ≥ 85 years [3]. The risk of developing HZ is also elevated among people who are immunodeficient or immunosuppressed due to disease or therapy [4, 5] (hereafter referred to collectively as “immunocompromised” [IC]). For example, annual HZ incidences of 9 per 1000 adults with human immunodeficiency virus (HIV) [6], 12 per 1000 adults with solid tumors [7], 24 per 1000 adults after kidney transplantation [8], 31 per 1000 adults with hematologic malignancies [7], and 60 per 1000 adults who have undergone autologous hematopoietic cell transplant [9] have been reported. Healthcare resource use and costs per case of HZ among IC versus immunocompetent patients have also been shown to be considerably higher, in both the United States (US) [10] and England [11].

Canada’s National Advisory Committee on Immunization (NACI) [1, 12] and Quebec’s Comité sur l’Immunisation du Québec (CIQ) [13] recommend recombinant zoster vaccine (RZV, Shingrix) for people aged ≥ 50 years without contraindications. Quebec’s CIQ also recommends RZV for IC patients aged ≥ 18 years [13], and while the NACI recommendations have not been updated to reflect the recent RZV indication, NACI currently advises that RZV may be considered for IC adults aged ≥ 50 years and state they will monitor and reassess recommendations for IC patients as evidence becomes available [1, 12]. RZV has been shown to be effective in patients who had undergone autologous stem cell transplantation [14] and those with potential immune-mediated diseases [15], and immunogenic in patients who had undergone renal transplantation [16] and those with HIV [17], hematological malignancies [18], or solid tumors [19].

Prior studies have estimated that RZV is likely to be a cost-effective option for the prevention of HZ among the general population of adults aged ≥ 50 years in Canada [20, 21]. The cost-efficacy of RZV among IC adults in Canada has not yet been assessed, but two recent studies have examined the cost-effectiveness of RZV in IC adults in the US [22, 23]. One considered various classes of IC adults aged 19–49 years and estimated that RZV would be cost saving for hematopoietic stem cell transplant (HSCT) recipients and patients with multiple myeloma, and to have incremental cost-effectiveness ratios (ICERs) ranging from $9500 per quality-adjusted life-year (QALY) gained for patients with hematologic malignancies to $208,000 per QALY gained for those with selected autoimmune/inflammatory conditions [22]. The other study considered various subgroups of IC adults with various starting ages (ranging from 25 to 45 years) and estimated that RZV would be cost saving for HSCT and renal transplant recipients, and to have ICERs ranging from $33,268 for people with HIV to $95,972 for patients with Hodgkin lymphoma per QALY gained [23]. The current analyses were conducted to estimate the cost-effectiveness and public health impact of RZV for selected populations of IC adults in Canada.

## Methods

### Model overview

**Fig. 1:**
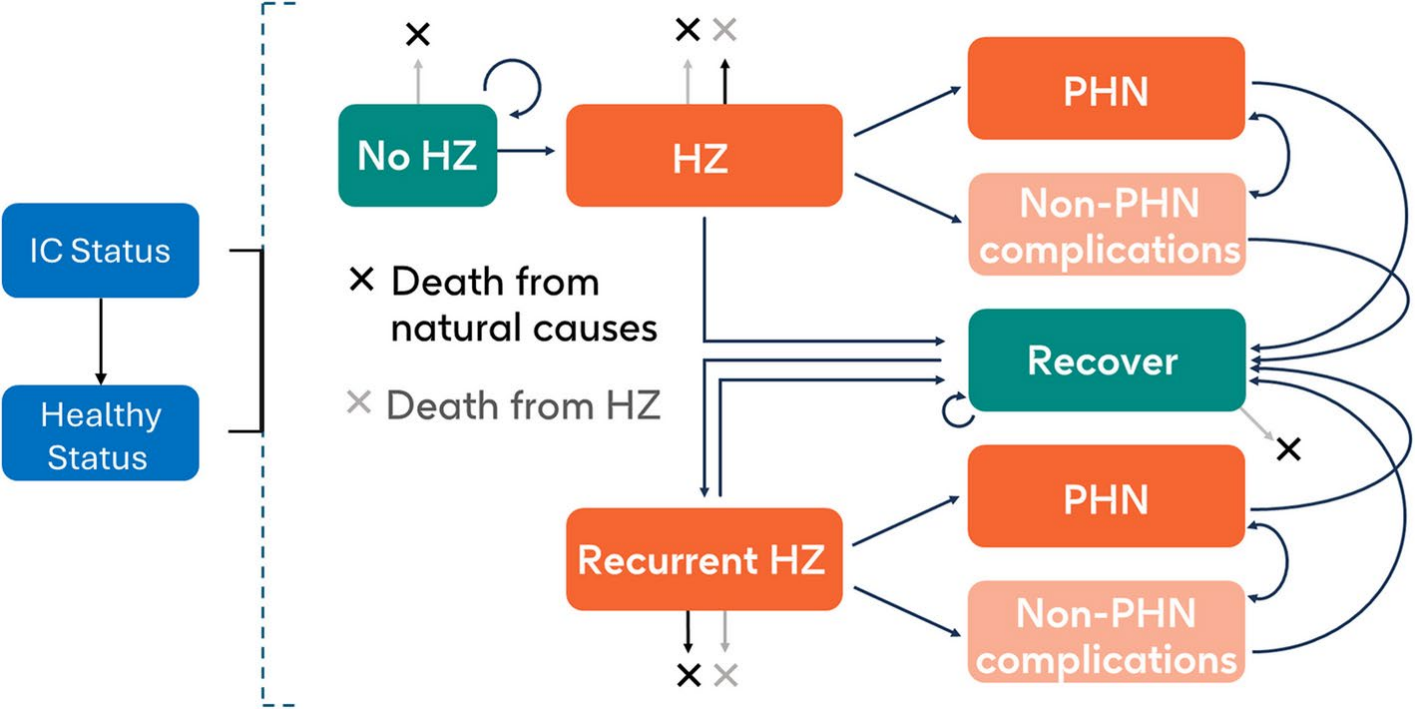
Underlying structure of the ZONA IC model. ^a^Modified from Curran et al. [24]. ^a^Vaccination may or may not occur in the “No HZ” health state, depending on the modeled strategy. Blue boxes indicate overall health states; dark and light orange boxes indicate initial and recurrent HZ states, respectively; green boxes indicate no HZ states. HZ, herpes zoster; IC, immunocompromised; PHN, postherpetic neuralgia; ZONA IC ZOster ecoNomic Analysis ImmunoCompromised

Outcomes in this study were estimated using the ZOster ecoNomic Analysis ImmunoCompromised (ZONA IC) model, which has previously been used to study various IC populations in the US [23, 24]. The ZONA IC model was adapted from the ZONA model, a Markov model that has previously been used to estimate HZ outcomes among adults aged ≥ 50 years in Canada [20, 21] and elsewhere [25–33]. In the current analysis, the ZONA IC model was used to observe populations of Canadian IC adults who were either vaccinated with RZV (two doses 2 months apart) or received no HZ vaccine at the start of the simulation. RZV is the only HZ vaccine currently recommended for IC patients in Canada [1, 12, 13], therefore only RZV and no HZ vaccination strategies were considered. The ZONA IC model was programmed in Excel (Microsoft Corporation; Redmond, Washington). The underlying model structure was a single-cohort, Markov model with a cycle length of 1 year. An overview of the structure of the ZONA IC model is shown in Fig. 1.

The ZONA IC model observes HZ status and outcomes in patients who are initially IC but who may progress to a healthy status over time. Modelled individuals were assumed not to have previously received any HZ vaccination. They were assumed to have IC status for IC condition-dependent durations, after which they were assumed to have “healthy” status, and input parameters for immunocompetent populations were applied. In the model, “healthy” denotes HZ epidemiology, direct and indirect costs, utilities, and all-cause mortality rates that are the same as those for an immunocompetent population of the respective ages modeled. Modelled starting ages were based on IC-condition specific ages as detailed in the “Scenario analyses” (Methods) section. A lifetime time horizon was selected to capture all relevant outcomes of HZ and HZ vaccination. An annual discount rate of 1.5% was applied for all costs, life-years, and QALYs, as is recommended by Canada’s Drug and Health Technology Agency [34] and NACI [35]. Cost outcomes are reported in 2022 Canadian dollars and we assumed a cost-effectiveness threshold of $50,000 per QALY gained. Although not formally recommended by NACI, this threshold is commonly cited in appraisals [36].

Multiple IC populations were studied, including an HSCT population in the base case, and four other IC populations (patients with breast cancer [as an example of a solid tumor], renal transplant recipients, people living with HIV, and patients with Hodgkin lymphoma [as an example of a hematological malignancy]) in scenario analyses. The societal perspective (i.e., direct and indirect costs) was applied in the base case and the four other IC population scenario analyses. The health-sector perspective (for HSCT) was explored in a scenario analysis [37].

### Base-case study population

An HSCT population was considered in the base-case probabilistic analysis because these patients have a high HZ incidence [9] and sufficient data availability for RZV efficacy and safety [14], HZ utility loss [38, 39], and HZ cost [11] data. A cohort size of 1600 was considered, to be consistent with the HSCT rate in North America in 2016 (511 per 10 million adults) [40], which was applied to the adult population of Canada in 2022 [41]. The starting age was assumed to be 55 years to reflect the mean/median ages in two studies of HSCT recipients [9, 14]. IC status duration was assumed to be 5 years, which was the follow-up duration in a study by Sahoo et al. [9] that determined the incidence of HZ for 5 years after HSCT.

### Base-case input parameters

Model inputs were informed by clinical trial data (for RZV efficacy and waning [24]), the scientific literature (for epidemiology and utility inputs), and Canadian data sources (for cost inputs) where available. Assumptions (where necessary) were validated by external health economic and clinical experts (see Supplemental Text 1).

An annual probability of all-cause mortality for HSCT recipients of 0.1168 was used, to reflect 5-year survival estimates for patients with multiple myeloma, non-Hodgkin lymphoma, Hodgkin lymphoma, and acute myeloid leukemia [42] and the proportions of HSCT recipients with these conditions in the RZV phase 3 clinical trial by Bastidas et al. [14]. Epidemiological inputs relating to HZ incidence, fatality, PHN, and non-PHN complication rates (ocular, neurological, cutaneous, and other non-pain) for individuals with IC status are detailed in Supplemental Table 1 [2, 5, 9, 14, 20, 39, 43–45]. Case fatality rates for HZ in HSCT recipients were conservatively assumed to be the same as rates estimated for the general population [43].

First-dose coverage for RZV was assumed to be 60%, a value slightly higher than the 44% influenza vaccination coverage for adults aged 18–64 years with a chronic medical condition [46]. Second-dose completion was assumed to be 100%, as was assumed by Prosser et al. [47], being somewhat higher than the 81% completion of 4-dose HZ vaccine in Winston et al. [48]) (Table 1) [46, 49]. Efficacy and waning parameters for 2-dose RZV were estimated from the phase 3 clinical trial of RZV in HSCT recipients [14], and rates of adverse events (AEs) were from the same study.

**Table 1:** Vaccine-related inputs for the probabilistic base-case and sensitivity analyses

**Category/input**	**Base case [source]**	**Range**^**a**^** [source]; SE**
First-dose coverage^b^ %	60 [assumed]	50–80 [assumed]; NA
Second-dose compliance^c^ %	100 [assumed]	85 [48] to 100 [assumed]; NA
Initial RZV efficacy against HZ (2 doses)^d^ %	72.5 [24]	58.3–82.4 [24]; 6.13
Initial RZV efficacy against HZ (1 dose), %	58.02 [80% of 2-dose efficacy]^e^	46.42–69.63 [± 20%]; 5.92
Initial RZV efficacy against PHN (2 doses)^f^ %	94.82 [derived from data on file]	58.33–100 [derived from data on file]; 10.63
Initial RZV efficacy against PHN (1 dose), %	75.86 [80% of 2-dose efficacy]^e^	60.69–91.03 [± 20%]; 7.74
Annual waning of RZV efficacy (2 doses)^f,g^ %	9.10 [estimated from data on file]	4.55 [–50%] to 18.20 [+ 100%]; 3.48
Annual waning of RZV efficacy (1 dose)^g^ %	18.20 [200% of 2-dose waning]^e^	9.10–27.30 [± 50%]; 4.64
Incidence of AEs per RZV dose for ages 18–49 and ≥ 50 years, respectively^f^		
Local/general	0.845 and 0.827 [data on file]	Not varied
Primary care provider	0.0203 and 0.017 [data on file]	Not varied
Emergency room	0.0045 and 0 [data on file]	Not varied
Hospitalization	0.0045 and 0.0022 [data on file]	Not varied

*AE* adverse event, *CI* confidence interval, *HZ* herpes zoster, *IC* immunocompromised, *NA* not applicable, *PHN* postherpetic neuralgia, *RZV* recombinant zoster vaccine, *SE* standard error

^a^For the probabilistic base-case analysis, the uniform distribution was used for first-dose coverage and second-dose compliance; the beta distribution was assumed for all efficacy and waning-related inputs. The SEs applied in the beta distribution parameters were consistent with the reported ranges representing 95% CIs

^b^Base case was assumed to be higher than the 44% influenza vaccination coverage for adults aged 18–64 years with a chronic medical condition from [46]; lower bound was assumed to be 50% based on the coverage for pneumonia vaccine among people aged ≥ 65 years in Canada [49]; upper bound was assumed to be 80% to reflect national coverage goals for influenza vaccination [46]

^c^Base case was assumed to be 100% based on the assumption used in [47] and 81% completion of 4-dose HZ vaccine in [48]

^d^Year 1 RZV efficacy (95% CI) [data on file] was taken from the study reported in [14], as used by [24]

^e^Limited data were available to estimate efficacy and waning for 1-dose RZV due to high second-dose completion rates in [14]. Therefore, the efficacy of 1-dose RZV was assumed to be 80% that of 2-dose RZV, and annual waning of efficacy for 1-dose RZV was assumed to be 200% that of 2-dose RZV

^f^From [data on file] of the study reported in [14]

^g^These inputs apply to individuals with IC status only. Values for individuals with healthy status are detailed in Supplemental Table 6

The RZV cost in Canada was $130.14 per dose (the IMS 2022 Canadian list price) and the administration costs (including indirect costs for work time lost) varied by age and dose as shown in Supplemental Table 2 [50–55]. Direct and indirect costs for treating RZV AEs [50, 52–56] were also included in the vaccination costs. Age-dependent direct medical costs of treating HZ with or without PHN among individuals of IC status are detailed in Supplemental Table 2. Limited Canada-specific data were available to estimate the costs of HZ cases in IC populations, so ratios of HZ costs for IC populations to immunocompetent populations in England [11] were applied to Canadian HZ costs for healthy adults [44, 50, 53, 54, 57, 58] to estimate costs of HZ cases for HSCT recipients in Canada. Indirect HZ costs were also accounted for, as detailed in Supplemental Table 2 [39, 48, 53, 54]. All cost inputs that were not originally reported in 2022 Canadian dollars were converted to that cost-year using the health and personal care component of the Canadian Consumer Price Index (CPI) for direct medical costs and the CPI for all items for indirect costs [53, 54].

Baseline utilities were obtained from pre-HZ EuroQol 5 dimensions (EQ-5D) utilities for adult HSCT recipients [38] (Supplemental Table 3). QALY losses for HZ cases (unvaccinated/vaccinated and with/without PHN) among individuals with IC status were estimated from clinical trial data for HZ vaccines in HSCT recipients [14, 38, 39, 59] and other published sources [60, 61]. QALY losses due to RZV AEs were obtained from a previous economic analysis for HZ vaccination [62] based on data from a randomized controlled study [63].

Five years after HSCT, the population was assumed to convert to “healthy” status, and different inputs were used, as detailed in Supplemental Tables 4–8 [2, 20, 25, 31, 41, 44, 50, 51, 54, 55, 57, 58, 64–73].

### Probabilistic base-case analysis

Model outcomes were cases of HZ, PHN, and other complications; HZ-related deaths; costs (vaccination, direct and indirect HZ costs); life-years and QALYs; and numbers needed to vaccinate (NNV) to prevent one case of HZ and one case of PHN for the HSCT population. These were generated by performing 5000 Monte Carlo simulations, in which the values of all inputs in Table 1 and Supplemental Tables 1–3 and 5–8 except RZV price were simultaneously sampled from probability distributions. The IC status duration and cost parameters were sampled across gamma distributions. Second-dose compliance for RZV was sampled across a uniform distribution since it was set to 100% in the base case. All other parameters were sampled across beta distributions. The distribution parameters were determined to be consistent with the ranges around the inputs (as specified in Table 1 and Supplemental Tables 1–3 and 5–8) representing 95% confidence intervals. The outcomes from the RZV and no HZ vaccine strategies were then compared to calculate ICERs for the cost per QALY gained.

### Deterministic sensitivity analysis

A deterministic sensitivity analysis was conducted to estimate the sensitivity of the ICERs of the RZV versus no HZ vaccine strategies for the HSCT population to each of the model input values when varied from the mean values of the distributions applied in the base-case analysis. Each of the model inputs (or groups of related inputs, e.g., the same parameter among IC and healthy populations) were varied individually across the ranges shown in Table 1 and Supplemental Tables 1–3 and 5–8. IC status duration was varied from 2 to 5 years, based on the follow-up durations in three studies [9, 14, 48].

### Scenario analyses

A set of probabilistic scenario analyses were performed to consider the impact of differences in key epidemiological and vaccine characteristics of four other IC populations (breast cancer, renal transplant, HIV, and Hodgkin lymphoma) on ICERs and NNVs. The population starting ages were 60 years for breast cancer [42] and 55 years for renal transplant [74] based on Canadian statistics, 40 years for HIV based on a Canadian study [75], and 25 years for Hodgkin lymphoma based on US data [76]. IC status duration was either 2 years (breast cancer, Hodgkin lymphoma) [7] or the remainder of life (renal transplant, HIV) (Table 2). All-cause mortality [42, 74, 75], HZ incidence [6–8], and vaccine efficacy inputs [6–8, 14, 18, 77, 78] for the four conditions considered in the scenario analyses are detailed in Table 2, while direct HZ costs are shown in Supplemental Table 9 [11, 44, 50, 51, 54, 55, 57, 58, 71]. There are no clinical trials from which to extract vaccine efficacy and waning data in these conditions, so regression functions were developed based on the associated between HZ incidence in the placebo arm of the RZV clinical trials and efficacy and waning estimates from healthy and IC populations in those same trials [14, 18, 77, 78]. These were then used to estimate RZV efficacy and waning for patients with each of these conditions as a function of their HZ incidence. Please see Supplemental Text 2 and 3 for further details. All other inputs for these scenario analyses were assumed to be the same as in the base case, followed (as necessary) by the healthy inputs.

**Table 2:** Inputs for individuals with non-HSCT IC status for the probabilistic scenario analyses

	**Breast cancer**	**Renal transplant**	**HIV**	**Hodgkin lymphoma**
Population starting age, years	60 [42]^a^	55 [74]	40 [75]	25 [76]^b^
IC status duration, years	2 [7]^c^	Remaining life [expert opinion]	Remaining life [assumed]^d^	2 [7]^c^
Annual probability of all-cause mortality^e^	0.0252 [42]	0.0305 [74]	0.0233 [75]	0.0297 [42]
Annual incidence of initial and recurrent HZ, per person-year	0.0171 [7]^f^	0.0244 [8]	0.0093 [6]	0.0336 [7]^f^
Initial 2-dose RZV efficacy against HZ^g^ %	96.50	94.51	98.65	87.20
Initial 2-dose RZV efficacy against PHN^g^ %	97.21	96.99	97.45	96.70
Annual waning of 2-dose RZV efficacy^g^ %	4.11	5.15	2.33	6.09

*HIV* human immunodeficiency virus, *HSCT* hematopoietic stem-cell transplant, *HZ* herpes zoster, *IC* immunocompromised, *PHN* postherpetic neuralgia, *RZV* recombinant zoster vaccine

^a^Based on 49.7% of projected new cases of breast cancer in 2019 being among ages 50–69 years from Table 1.3 of [42]

^b^Based on the most common age group for new cases of Hodgkin lymphoma being 20–34 years [76]

^c^Based on the follow-up duration in [7], from which HZ incidence was taken

^d^Based on the inclusion of all people receiving primary care for HIV in the estimate of HZ incidence from [6]

^e^These values (which were estimated based on mortality rate [HIV] or 5-year survival rates [other conditions] using data from the sources shown) were applied until the population returned to healthy status or reached an age at which all-cause mortality for the general population was greater than the annual probability of all-cause mortality shown

^f^Based on HZ incidence rates for 12, 6, and 6 months of high/very high, moderate, and none/low immunosuppression, respectively

^g^Values were estimated from regression equations reflecting the association between placebo HZ incidence and RZV efficacy and waning in immunocompetent and IC populations from [14, 18, 77, 78]. The placebo HZ incidence estimates used to estimate efficacy and waning from the regression equations for the IC conditions were derived from [7] for breast cancer and Hodgkin lymphoma, [8] for renal transplant, and [6] for HIV. One-dose efficacy estimates were 80% of 2-dose estimates; 1-dose waning estimates were twice 2-dose waning estimates

Additionally, the health-sector perspective – which considers only direct costs – was considered for the HSCT population in a deterministic scenario analysis. In this scenario, indirect costs resulting from working hours lost due to RZV vaccination and AEs, HZ, and PHN were excluded.

Lastly, a set of deterministic scenario analyses were conducted to observe ICERs when the IC status duration, the annual HZ incidence during IC status, and the starting age of the model population were simultaneously varied. For further details, please see Supplemental Text 4.

## Results

### Base case

The results of the base-case analysis of RZV versus no HZ vaccine report outcomes for a cohort of 1600 adults aged 55 years in Canada who have recently received HSCT. RZV was predicted to prevent medians of 116 HZ cases, 27 PHN cases, and 14 non-PHN complications (Table 3). The median NNVs (90% uncertainty intervals) to avoid one HZ and one PHN case were 8 (5–15) and 35 (22–72), respectively.

**Table 3:** Base-case probabilistic analysis results for 1600 HSCT adults aged 55 years

	**RZV, median (90% UI)**	**No HZ vaccine, median (90% UI)**	**Incremental, median**^**a**^** (%)**
Health outcomes			
HZ cases	424 (326–540)	545 (430–677)	–116 (–21)
PHN cases	76 (56–103)	104 (79–134)	–27 (–26)
Complications	54 (39–76)	68 (49–94)	–14 (–20)
Ocular	18 (11–28)	22 (13–35)	–4 (18)
Neurological	13 (7–21)	15 (8–26)	–2 (–14)
Cutaneous	17 (9–27)	23 (13–37)	–6 (–26)
Other non-pain	6 (3–11)	7 (3–14)	–1 (–14)
HZ-related deaths	0 (0–0)	0 (0–0)	0
Discounted costs, $			
Vaccination costs^b^	358,214 (279,629–439,918)	0 (0–0)	358,214 (NA)
Direct HZ costs	396,949 (273,446–564,813)	574,104 (389,289–822,020)	–173,025 (–30)
Indirect HZ costs	113,887 (87,794–144,927)	144,709 (114,603–180,273)	–30,061 (–21)
Total societal costs^c^	872,228 (718,272–1,056,695)	719,906 (513,189–993,583)	148,491 (21)
Discounted life-years	22,211 (22,210–22,211)	22,211 (22,210–22,211)	0
Discounted QALYs	19,875 (17,385–21,292)	19,870 (17,378–21,285)	7 (0)
ICER,^d^ $ per QALY gained	NA	NA	22,648 (NA)

All costs are in 2022 Canadian dollars

*AE* adverse event, *HSCT* hematopoietic stem-cell transplant, *HZ* herpes zoster, *ICER* incremental cost-effectiveness ratio, *NA* not applicable, *PHN* postherpetic neuralgia, *QALY* quality-adjusted life-year, *RZV* recombinant zoster vaccine, *UI* uncertainty interval

^a^Median incremental outcomes are reported, which may not match the incrementals of the median outcomes from each vaccination scenario

^b^Vaccination costs include direct and indirect costs of vaccine acquisition and AEs

^c^Median total societal costs are reported, which may not match the sums of the medians of each category of costs

^d^From a societal perspective

**Fig. 2:**
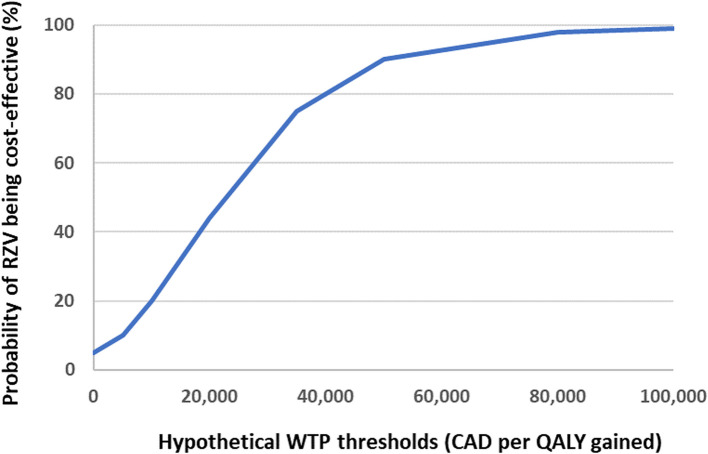
CEAC from 5000 simulations for RZV versus no HZ vaccine for Canadian HSCT adults. All costs are in 2022 Canadian dollars. CEAC, cost-effectiveness acceptability curve; HSCT, hematopoietic stem-cell transplant; HZ, herpes zoster; QALY, quality-adjusted life-year; RZV, recombinant zoster vaccine

Vaccination was predicted to have a median cost of $358,214, but this was predicted to be partly offset by savings of $173,025 in direct HZ costs and $30,061 in indirect HZ costs, resulting in a total median incremental societal cost of $148,491 over the lifetime time horizon (Table 3). This equated to a median (90% uncertainty interval) ICER of $22,648 ($205–$62,197) per QALY gained for RZV versus no HZ vaccine. Overall, 90.3% of the 5000 simulations resulted in an ICER ≤ $50,000 per QALY gained (Fig. 2 and Supplemental Fig. 1).

### Deterministic sensitivity analyses

**Fig. 3:**
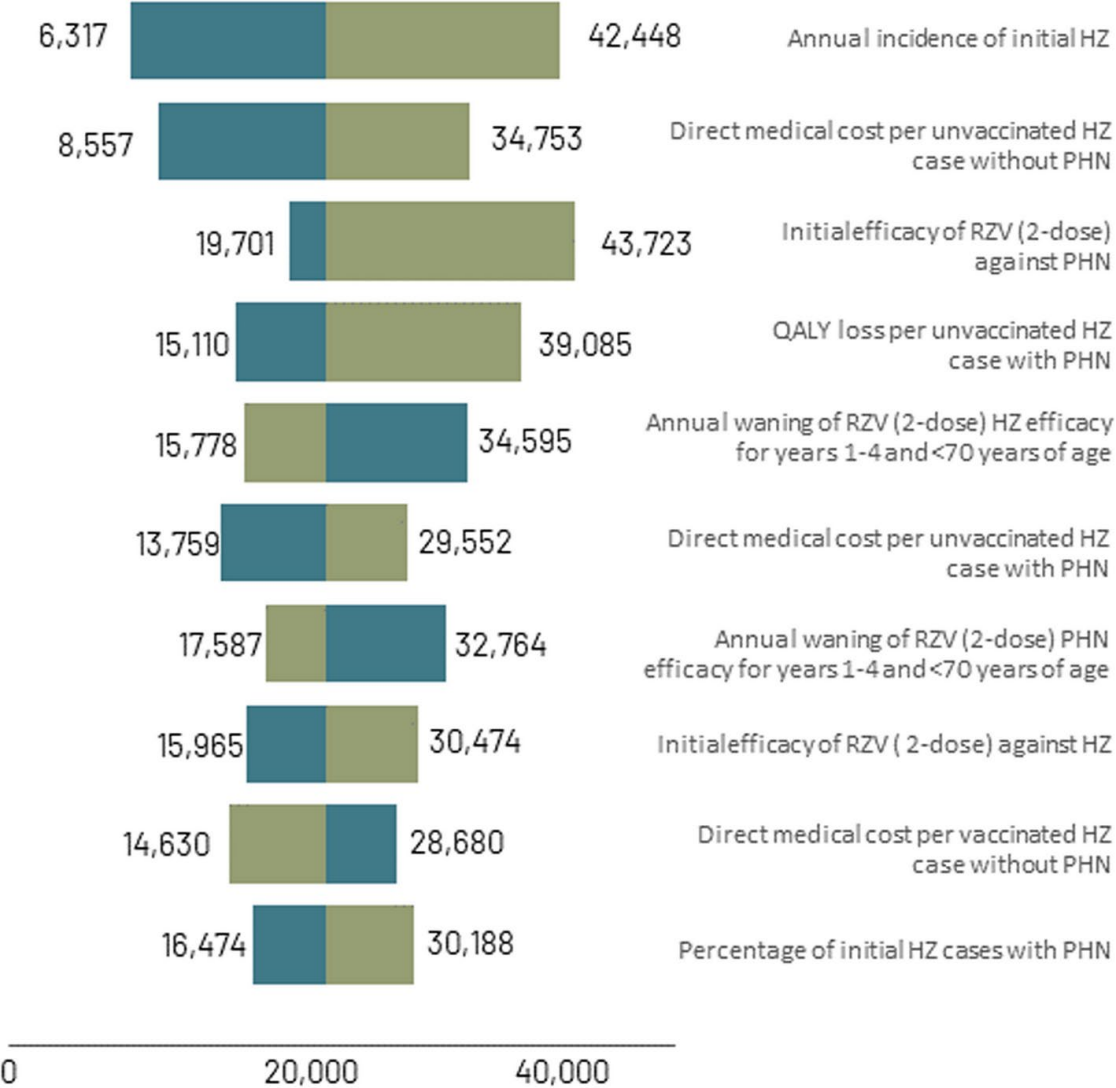
DSA results for the ICERs of RZV versus no HZ vaccine for Canadian HSCT adults^a^. All costs are in 2022 Canadian dollars. ^a^Only the top 10 results are shown. DSA, deterministic sensitivity analysis; HSCT, hematopoietic stem-cell transplant; HZ, herpes zoster; ICER, incremental cost-effectiveness ratio; PHN, postherpetic neuralgia; QALY, quality-adjusted life-year; RZV, recombinant zoster vaccine; YOA, years of age

The ICER was most sensitive to the annual incidence of initial HZ, direct medical cost per unvaccinated HZ case without PHN, initial efficacy of 2-dose RZV against PHN, and QALY loss per unvaccinated HZ case with PHN (Fig. 3). The highest ICERs ($43,723 and $42,448 per QALY gained) were observed when the initial efficacy of 2-dose RZV against PHN and the annual incidence of initial HZ, respectively, were at their lower bounds. These were still below the hypothetical cost-effectiveness threshold of $50,000 per QALY gained. Most input variations (87%) did not shift the ICER by more than $5000 in either direction.

### Scenario analyses

Probabilistic scenario median estimates of ICERs for IC conditions other than HSCT ranged from $24,328 per QALY gained for the breast cancer population to $81,470 per QALY gained for the Hodgkin lymphoma population (Table 4), the latter of which had the youngest starting age (25 years) and brief IC status durations (2 years) (Table 2). The median NNVs to avoid one HZ and one PHN case were 6–7 and 23–56, respectively, across IC conditions (Table 4).

**Table 4:** Scenario analysis results for RZV versus no HZ vaccine for selected Canadian adult IC populations

	**Median (90% UI)**		
	**ICER, $ per QALY gained**	**NNV to avoid**	
		**1 HZ case**	**1 PHN case**
Breast cancer scenario	24,328 (15,010–39,864)	6 (4–9)	23 (16–37)
Renal transplant scenario	27,237 (8631–58,779)	6 (4–9)	42 (27–71)
HIV scenario	67,207 (39,698–115,403)	7 (5–11)	56 (36–92)
Hodgkin lymphoma scenario	81,470 (38,262–179,274)	7 (4–17)	38 (19–88)

All costs are in 2022 Canadian dollars from a societal perspective

*HIV* human immunodeficiency virus, *HZ* herpes zoster, *IC* immunocompromised, *ICER* incremental cost-effectiveness ratio, *NNV* number needed to vaccinate, *PHN* postherpetic neuralgia, *QALY* quality-adjusted life-year, *RZV* recombinant zoster vaccine, *UI* uncertainty interval

When the ZONA IC model was used to estimate the cost-effectiveness of using RZV to vaccinate Canadian adults with recent HSCTs aged 55 years from a health-sector perspective, the deterministic health-sector ICER was predicted to be slightly lower than the median probabilistic societal perspective ICER, at $21,114 per QALY gained.

The results of the deterministic scenario analyses simultaneously varying IC status duration and annual HZ incidence for the cohorts with starting ages of 45, 35, and 25 years are presented in Supplemental Fig. 2. The ICERs ranged from $3010 (when each input was at its maximum) to $141,729 (when each input was at its minimum).

## Discussion

The results of this study suggest that RZV could reduce the public health burden of HZ for Canadian IC adults with selected IC conditions. Further, that RZV is likely to be cost-effective relative to no HZ vaccination (at an assumed $50,000 per QALY threshold) in HSCT recipients (base-case probabilistic median societal ICER: $22,648) and the base-case result was robust to a wide range of uncertainties in key parameters that influenced the benefits and costs of the vaccine program. The breast cancer and renal transplant scenarios also indicated that RZV would be cost effective in these populations, while ICERs for the HIV and Hodgkin lymphoma scenarios were above (but less than double) the assumed threshold. In additional scenario analyses, ICERs were highest when the population was younger, the HZ incidence during IC status was lower, and the IC status duration was shorter.

When the ZONA IC model was used to estimate the cost-effectiveness of using RZV to vaccinate Canadian adults with recent HSCT aged 55 years from a health-sector perspective, the deterministic ICER ($21,114 per QALY gained) was similar to the probabilistic societal perspective ICER $22,648 per QALY gained). Although it is difficult to compare these ICERs directly, given their different analysis methods, their similarity implies that the effect of excluding indirect costs (i.e., lost earnings) associated with RZV administration and AEs was similar to the effect of excluding indirect costs associated with HZ cases. Notably, AEs are more common in IC adults than in a general population of adults aged ≥ 50 years based on data on file from randomized controlled trials [14, 77, 78], so indirect costs associated with AEs were higher for IC adults. Also of note, indirect cost inputs per HZ case were much lower for IC adults relative to immunocompetent adults, partly due to the different methods used to estimate these inputs in the current study and a previous study in adults aged ≥ 50 years [20], but also potentially reflecting that fewer IC adults are employed.

To our knowledge, only two other studies have estimated the cost-effectiveness of RZV for IC adults, both in the US [22, 23]. The first of these used a time-step state transition model to predict that RZV would be cost-saving among HSCT recipients and patients with multiple myeloma, and would have QALYs of $9500 (hematologic malignancies), $79,000 (HIV), $165,000 (non-Hodgkin lymphoma), and $208,000 (selected autoimmune/inflammatory conditions) (2020 US dollars) among adults aged 19–49 years [22]. The other study was conducted using the ZONA IC model and included the same five populations as the current study, but with younger starting ages, a 30-year time horizon (rather than lifetime), and focused on adults aged 18–49 years [23]. The US ZONA IC model estimated that RZV would be cost saving for HSCT and renal transplant recipients, and to have ICERs of $33,268 (HIV), $67,682 (breast cancer), and $95,972 (Hodgkin lymphoma) per QALY gained (2019 US dollars) [23]. Another US study has examined the public health impact of RZV for populations aged ≥ 18 years with HSCT, breast cancer, or Hodgkin lymphoma [24]. That US study [24] predicted 38–60% reductions in HZ cases, 59–73% reductions in PHN cases, and 41–61% reductions in complications across the three IC conditions. The NNVs to prevent one HZ case were 8–10 across the three IC conditions, while the NNVs to prevent one PHN case were 47–91 [24].

As previously mentioned, Quebec’s CIQ already recommends RZV for IC patients aged ≥ 18 years [13], while NACI currently advises that RZV may be considered for IC adults aged ≥ 50 years on a cases-by-case assessment of the benefits compared to the risks [1, 12], preferably ≥ 14 days before starting immunosuppressive treatment [1]. Our findings may help to inform policy and decision makers on the value of HZ prevention in the Canadian IC population.

### Generalizability

The base-case analysis considered a population of adult HSCT recipients. This population represents a small portion of the overall IC population, including only approximately 1600 people estimated to receive HSCTs annually in Canada [40, 41]. Therefore, we conducted multiple scenario analyses to consider key population and epidemiological factors for various subsets of the heterogeneous conditions expected in the broader IC population.

As HSCT recipients are expected to have a higher underlying HZ risk than individuals in other IC populations [4, 5], the lower HZ risk in other IC populations would tend to reduce the cost-effectiveness of RZV vaccination if other factors remained equal. Indeed, the results of the scenario analyses in which the ICERs for patients with breast cancer, renal transplant, HIV, or Hodgkin lymphoma were higher than the ICERs for HSCT recipients, although these included other differences, including starting age, IC duration, etc.

HSCT recipients have a transient duration of IC status that may be longer or shorter than individuals in other IC populations. Duration of IC status is expected to be heterogeneous in the broader IC population, with some populations expected to experience long-term immune suppression (e.g., primary or acquired immunodeficiency, solid organ transplant recipients requiring lifelong immunosuppressive treatment) and other populations expected to experience peaks and troughs of elevated HZ risk as these individuals initiate and discontinue immunosuppressive treatment. The additional scenarios– in which starting age, IC status duration, and HZ incidence were varied–demonstrated that shorter IC duration had a much larger impact on ICERs in the youngest population because older populations experience age-related elevations in HZ risk regardless of IC status. If all other factors were equal, a longer IC duration would increase the favorability of RZV cost-effectiveness (and vice versa).

Available evidence suggests that RZV efficacy declines and waning increases with decreasing immune function [14, 18, 77, 78]. HSCT recipients, therefore, have a lower RZV efficacy and higher waning relative to the overall IC population and to healthy adults. As projected using regression functions, we would expect more favorable efficacy and waning for RZV in many IC populations and in the overall IC population than in the HSCT population; those factors would potentially improve the cost-effectiveness of RZV vaccination in those populations.

HSCT recipients have higher all-cause mortality rates than other IC populations studied [42, 74, 75]. More generally, we expect that patients with HSCT have higher mortality rates than many other IC populations, especially patients with autoimmune conditions, who comprise the majority of the IC population.

Sensitivity and scenario analyses demonstrated that several factors that are expected to vary across IC populations impact the predicted cost-effectiveness of vaccination with RZV, especially initial efficacy and waning rates, underlying HZ incidence, age at vaccination, and duration of IC status. Overall, the heterogeneity of IC patients makes it difficult to estimate the cost-effectiveness of RZV for the overall IC population.

### Limitations

This analysis made the simplifying structural assumption that the IC status duration was a discrete number of years and that the population maintained a healthy status for the remainder of the time horizon (i.e., the population did not have multiple IC periods). However, the IC duration and resulting HZ risk may be more dynamic in real-world IC populations. For example, adults with autoimmune diseases may experience multiple transient periods of immune suppression as they initiate and then stop immunosuppressive treatments over time. Therefore, multiple combinations of IC status duration and magnitudes of HZ risk during IC status were explored in scenario analyses.

We assumed RZV first-dose coverage of 60% and a 100% series completion rate but tested lower values in sensitivity analysis. Of note, first-dose coverage does not affect ICER estimates, and second-dose completion was shown to have a limited impact in sensitivity analysis.

Although we used Canada-specific estimates where possible, these were not always available. For example, HZ incidence among HSCT recipients was based on data from the United States. Further, direct HZ-related costs among IC patients were estimated based on direct HZ-related costs among healthy Canadian adults [44, 50, 53, 54, 57, 58] and the ratio of costs between IC and healthy adults in England [11]. Although we sought the advice of clinical and economic experts before applying non-Canadian inputs, we acknowledge that this approach could have resulted in over- or under-estimations of HZ incidences and HZ costs. We also note that, within the general Canadian population, there are regional, ethnic, and sex disparities in RZV coverage [79]. Such disparities may also be present among IC patients, limiting the generalizability of the analysis results.

In addition, limited data were available to estimate RZV efficacy and waning in IC populations other than HSCT recipients. Current understandings of RZV efficacy in other IC populations is limited to a post-hoc analysis in a population with hematologic malignancies [18]. To address this issue, regression analyses were conducted using data from RZV clinical trials in healthy and IC populations. For each IC population considered in scenario analysis, the regression analyses estimated potential differences in RZV characteristics as a function of the magnitude of HZ risk during IC status. A key limitation of the regression analysis is that it assumes that HZ incidence for an IC condition is a proxy to represent severity of relevant immune function impairment and completely explains variation in RZV efficacy and waning. Although this approach could have resulted in over- or under-estimates of RZV efficacy and waning, it was validated with external clinical and health economics experts and has been used in a similar modelling study [23]. Lastly, we considered a hypothetical cost-effectiveness threshold of $50,000 per QALY gained.

## Conclusions

These public health impact and cost-effectiveness results for selected IC populations and scenarios suggest that RZV could reduce the burden of HZ and its associated complications for Canadian IC adults. RZV is likely to be considered cost-effective relative to no HZ vaccination in HSCT recipients and various other IC populations.

## Supplementary Information

Supplementary Material 1.

## Data Availability

No datasets were generated or analysed during the current study.

## Abbreviations

AE: Adverse event
CIQ: Comité sur l’Immunisation du Québec
CPI: Consumer Price Index
EQ-5D: EuroQol 5 dimensions
HIV: Human immunodeficiency virus
HSCT: Hematopoietic stem cell transplant
HZ: Herpes zoster
IC: Immunocompromised
ICER: Incremental cost-effectiveness ratio
NACI: National Advisory Committee on Immunization
NNV: Number needed to vaccinate
PHN: Postherpetic neuralgia
QALY: Quality-adjusted life-year
RZV: Recombinant zoster vaccine
US: United States
ZONA IC: ZOster ecoNomic Analysis ImmunoCompromised
