# Supplementary material for: Cost-effectiveness and public health impact of recombinant zoster vaccine versus no herpes zoster vaccination in selected populations of immunocompromised adults in Canada
Source: BMC Health Serv Res. 2025 Apr 25;25:604. doi: 10.1186/s12913-025-12550-x (PMC12023514; doi:10.1186/s12913-025-12550-x)
Supplement: Supplementary file 1 — Supplementary Material 1. [file 12913_2025_12550_MOESM1_ESM.docx]

Supplementary material for Cost-effectiveness and public health impact of recombinant zoster vaccine versus no herpes zoster vaccination in selected populations of immunocompromised adults in Canada

*BMC Health Serv Res*

Sydney George^1*^, Justin Carrico^2^, Katherine A Hicks^2^, Dessi Loukov^1^, Cheryl Ng^3^, Desmond Curran^4^

^1^GSK, Mississauga, Ontario, Canada. ^2^RTI-Health Solutions, Research Triangle Park, North Carolina, US. ^3^GSK, Singapore, Singapore. ^4^GSK, Wavre, Belgium

## Contents

**Supplemental Text 1** Validation

**Supplemental** **Text 2** Methodology for estimating initial two-dose efficacy

**Supplemental** **Text 3** Methodology for estimating waning efficacy

**Supplemental Text 4** Scenario analyses

**Supplemental Table 1** Epidemiological inputs for the probabilistic base-case and sensitivity analyses

**Supplemental Table 2** Cost inputs for the probabilistic base-case and sensitivity analyses

**Supplemental Table 3** Utility inputs for the probabilistic base-case and sensitivity analyses

**Supplemental Table 4** Annual probabilities of all-cause mortality for people with healthy status

**Supplemental Table 5** Epidemiological inputs for people with healthy status

**Supplemental Table 6** Waning inputs for people with healthy status

**Supplemental Table 7** Direct HZ cost inputs for people with healthy status

**Supplemental Table 8** Utility inputs for people with healthy status

**Supplemental Table 9** Direct HZ cost inputs for people with non-HSCT IC

**Supplemental Fig. 1** Incremental costs versus incremental QALYs from 5000 probabilistic base-case analysis simulations of RZV versus no HZ vaccine for Canadian HSCT adults aged 55 years

**Supplemental Fig. 2** ICERs for RZV versus no HZ vaccine for select populations of Canadian immunocompromised adults aged (a) 45 years, (b) 35 years, and (c) 25 years at model start, across ranges of values for the IC status duration and the annual HZ incidence

**Supplemental references**

**Supplemental Text 1** Validation

External health economic and clinical experts were consulted to gain insight on the approach, inputs, and assumptions of the model for which published data were limited or not available. The inputs with limited Canada-specific data for which external experts were consulted for validation included the annual herpes zoster (HZ) incidence for hematopoietic stem cell transplant (HSCT) recipients and costs of HZ cases for immunocompromised (IC) adults. The inputs for which experts were consulted for validation due to limited published data included recombinant zoster vaccine (RZV) efficacy and waning for other modeled IC populations (human immunodeficiency virus [HIV], renal transplant, Hodgkin lymphoma, and breast cancer), indirect costs associated with vaccine administration, and indirect costs of vaccine-related adverse events.

Regarding the HZ incidence in HSCT recipients, Su et al. [1] reported an HZ incidence of 175 per 1,000 person-years among 179 HSCT recipients in Montreal, Canada. However, the study population received no antiviral prophylaxis for varicella zoster virus during the study period, and the incidence reported in this study is significantly higher than other estimates of HZ incidence in HSCT recipients in the literature. Therefore, the mean annual HZ incidence for the base-case analysis was obtained from an epidemiological study of HZ incidence among autologous HSCT recipients in the United States (US), which reported 60 HZ cases per 1,000 person-years in the 5 years following transplant [2]. Clinical expert opinion was solicited to assess the validity of applying a US-specific incidence estimate to Canadian HSCT recipients. Per expert opinion, clinical treatment of Canadian HSCT recipients is similar to those in the US because Canada uses US protocols for the treatment of HSCT recipients. Nonetheless, a wide range informed by a targeted, global literature review of HZ incidence estimates in HSCT recipients was used in a sensitivity analysis to consider the uncertainty associated with applying non-Canada-specific data.

As no Canadian studies were available to estimate HZ costs in IC adults, HZ cost estimates from an observational retrospective database analysis in England were used to derive age-specific ratios of HZ costs for IC populations to HZ costs for an immunocompetent population [3]. Ratios were than multiplied by Canadian HZ cost estimates for immunocompetent adults [4-9] to estimate HZ costs in IC adults. This derivation approach was validated with external health economic experts, who commented that the approach would have additional validity if healthcare resource utilization were similar between the United Kingdom (UK) and Canada. Although limited data are available to compare healthcare resource utilization between the UK and Canada, healthcare spending as a percentage of gross domestic product is similar in the two countries (11.9% for the UK and 11.7% for Canada in 2021) [10].

As limited data were available to estimate RZV effectiveness and durability in IC populations other than HSCT, regression analyses were conducted using data from RZV clinical trials in healthy and IC populations (see Supplementary Texts 2 and 3). For each IC population considered in scenario analyses, the regression analyses estimated potential differences in RZV characteristics as a function of the magnitude of HZ risk during IC status. A key limitation of the regression analysis is that it assumes that HZ incidence for an IC condition is a proxy to represent severity of relevant immune function impairment and completely explains variation in RZV efficacy and waning. The approach was validated with external clinical and health economics experts.

**Supplemental** **Text 2** Methodology for estimating initial two-dose efficacy

Data are not currently available for the efficacy of recombinant zoster vaccine (RZV) in human immunodeficiency virus (HIV), renal transplant, and breast cancer populations. Data from RZV's clinical trials in healthy and immunocompromised (IC) populations suggest that vaccine efficacy is lower in populations with higher placebo herpes zoster (HZ) incidence (i.e., immunosuppression) (Table A).

**Table A.** RZV efficacy decreases with increasing HZ incidence

| Clinical trial | Population | HZ incidence among unvaccinated (per 1,000 PY) | Initial RZV efficacy, % | |
| --- | --- | --- | --- | --- |
|  |  |  | HZ | PHN |
| ZOE-50 [11] | Age ≥50 years | 9.1 | 98.4 | 100.0 |
| ZOE-70 [12] | Age ≥70 years | 9.3 | 97.6 | 94.9 |
| ZOE-HM [13] | HM patients | 66.2 | 87.2^a^ | – |
| ZOE-HSCT [14] | HSCT recipients | 94.3 | 72.5 | 94.8 |

HM, hematological malignancy; HSCT, hematopoietic stem cell transplantation; HZ, herpes zoster; PHN, post-herpetic neuralgia; PY, person-years.

^a^Post-hoc efficacy analysis.

Based on the above data, linear regression functions were developed to estimate initial efficacy of RZV against HZ and PHN based on HZ incidence among unvaccinated individuals (Figure A). A limitation of this regression methodology is that it does not consider predictive immunological factors other than HZ incidence among unvaccinated individuals that may contribute to differences in RZV effectiveness between the modeled populations.

**Figure A.** Association between HZ incidence in the placebo arms and initial RZV efficacy against HZ and PHN [11-14].


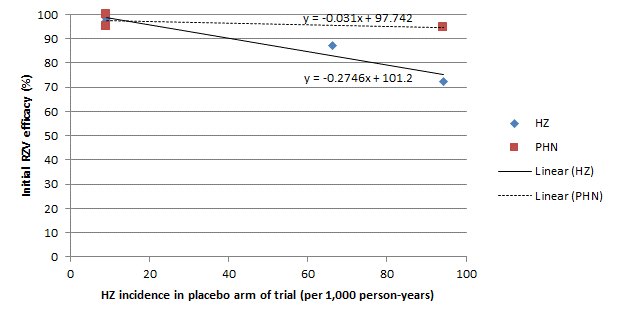


Using the above-derived equations, HZ incidences among patients with HIV, renal transplant, and breast cancer were used to estimate initial RZV efficacy as detailed in Table B.

**Table B.** Initial 2-dose RZV efficacies calculated from HZ incidences

|  | HIV | Renal transplant | Breast cancer |
| --- | --- | --- | --- |
| Placebo HZ incidence, per 1,000 PY [ref] | 9.3^a^ [15] | 24.4^b^ [16] | 17.1^c^ [17] |
| Initial 2-dose HZ efficacy,^d^ % | 98.65 | 94.51 | 96.50 |
| Initial 2-dose PHN efficacy,^e^ % | 97.45 | 96.99 | 97.21 |

**^a^**HIV patients at an urban HIV clinic between 2002 and 2009.

^b^Patients who received kidney transplants between 1995 and 2007.

^c^Adults with solid tumors from 2001 to 2006.

^d^Estimated as (–0.2746 × [placebo HZ incidence]) + 101.2.

^e^Estimated as (–0.031 × [placebo HZ incidence]) + 97.742.

**Supplemental** **Text 3** Methodology for estimating waning efficacy

Data are not currently available for the waning of recombinant zoster vaccine (RZV) efficacy in human immunodeficiency virus (HIV), renal transplant, Hodgkin lymphoma, and breast cancer populations. Data from RZV clinical trials in healthy and immunocompromised (IC) populations suggest that vaccine waning is higher in populations with higher placebo herpes zoster (HZ) incidence (i.e., immunosuppression) (Table A).

**Table A.** RZV waning increases with increasing HZ incidence

| Clinical trial | Population | HZ incidence among unvaccinated (per 1,000 PY) | Annual waning of RZV efficacy, % |
| --- | --- | --- | --- |
| ZOE-50 [11] | Age ≥50 years | 9.1 | 1.0 |
| ZOE-70 [12] | Age ≥70 years | 9.3 | 3.6 |
| ZOE-HSCT [14] | HSCT recipients | 94.3 | 9.1 |

HSCT, hematopoietic stem cell transplantation; HZ, herpes zoster; PY, person-years.

Based on the above data, logarithmic regression functions were developed to estimate waning of two-dose RZV efficacy against HZ and PHN based on HZ incidence among unvaccinated individuals (Figure A). A limitation of this regression methodology is that it does not consider predictive immunological factors other than HZ incidence among unvaccinated individuals that may contribute to differences in RZV effectiveness between the modeled populations.

**Figure A.** Association between HZ incidence in placebo arms and waning of RZV efficacy against HZ and PHN [11-14].

Using the above-derived equations, HZ incidences among patients with HIV, renal transplant, Hodgkin lymphoma, and breast cancer were used to estimate RZV waning as detailed in Table B.

**Table B.** Annual waning of RZV efficacies calculated from HZ incidences

|  | HIV | Renal transplant | Hodgkin lymphoma | Breast cancer |
| --- | --- | --- | --- | --- |
| Placebo HZ incidence, per 1,000 PY [ref] | 9.3^a^ [15] | 24.4^b^ [16] | 33.6^c^ [17] | 17.1^d^ [17] |
| Annual waning after 2 doses,^e^ % | 2.33 | 5.15 | 6.09 | 4.11 |
| Annual waning after 1 dose,^f^ % | 4.65 | 10.29 | 12.17 | 8.22 |

**^a^**HIV patients at an urban HIV clinic between 2002 and 2009.

^b^Patients who received kidney transplants between 1995 and 2007.

^c^Adults with hematologic malignancies from 2001 to 2006.

^d^Adults with solid tumors from 2001 to 2006.

^d^Estimated as (2.924 × ln[placebo HZ incidence]) – 4.2068.

^e^Estimated as twice the waning after 2 doses.

**Supplemental Text 4** Scenario analyses

Immunocompromised (IC) status durations (1, 2, 3, 4, 5, and 30 years) were varied to consider transient or lifelong immunosuppression. Annual herpes zoster (HZ) incidences (10, 20, 30, 40, 50, 60, 70, and 80 per 1000 person-years) were assumed to reflect the spectrum of HZ risk observed across IC conditions [18]. Each combination of IC status duration and annual HZ incidence was explored for population cohorts with starting ages of 45, 35, and 25 years. Age-specific annual probabilities of all-cause mortality were assumed to be twice the probability of immunocompetent individuals to reflect IC populations having an underlying risk for death that is greater than the general Canadian population but is less than the hematopoietic stem cell transplant (HSCT) population modeled in the base-case analysis. All other input values were consistent with the mean values applied in the base-case analysis.

**Supplemental Table 1** Epidemiological inputs for the probabilistic base-case and sensitivity analyses

| **Category/input** | **Base case [source]** | **Range^a^ [source]; SE** |
| --- | --- | --- |
| Annual incidence of initial and recurrent HZ,^b^ per person-year | 0.06 [2] | 0.0402 [19] to 0.09428 [14]; 0.0138 |
| Case fatality rates for HZ cases,^c^ % |  |  |
| Age 18–64 years | 0 [assumed] | 0–0 [assumed]; 0 |
| Age 65–69 years | 0.012 [20] | 0.0027–0.083 [5, 20-23]; 0.0205 |
| Age 70–74 years | 0.012 [20] | 0.0035–0.083 [5, 20-23]; 0.0203 |
| Age 75–84 years | 0.076 [20] | 0.0078–0.083 [5, 20-23]; 0.0192 |
| Age ≥ 85 years | 0.076 [20] | 0.0078–0.2016 [5, 20-23]; 0.0494 |
| Initial and recurrent HZ cases with PHN,^b,d^ % | 12.89 [2] | 8.51–17.27 [2]; 2.23 |
| Initial and recurrent HZ cases with HZ-related complications,^b,e^ % |  |  |
| Ocular | 3.56 [2] | 1.14–5.98 [2]; 1.23 |
| Neurological | 1.78 [2] | 0.05–3.50 [2]; 0.88 |
| Cutaneous | 5.78 [2] | 2.73–8.83 [2]; 1.56 |
| Other non-pain | 0.89 [2] | 0.00–2.12 [2]; 0.63 |

^a^ For the probabilistic base-case analysis, the beta distribution was assumed for the purpose of sampling values for all inputs in this table. The SEs applied in the beta distribution parameters are consistent with the ranges representing 95% CIs

^b^ These inputs apply to individuals with IC status only. Values for individuals with healthy status are shown in Supplemental Table 5

^c^ Assumed to be the same as for a healthy population

^d^ Range was derived from [2] based on number of events and sample size

^e^ The complication rates were only used for the generation of health outcomes; no costs due to complications other than PHN were explicitly considered to avoid potential double-counting with costs related to HZ from [24]

CI, confidence interval; HZ, herpes zoster; IC, immunocompromised; PHN, postherpetic neuralgia; SE, standard error

**Supplemental Table 2** Cost inputs for the base-case and sensitivity analyses

|  | **Base case (range^a^); SE** | | | | |
| --- | --- | --- | --- | --- | --- |
|  | **Age 18–49 years** | **Age 50–59 years** | **Age 60–64 years** | **Age 65–69 years** | **Age ≥ 70 years** |
| RZV per dose, $ (IMS) | 130.14 (not varied) | 130.14 (not varied) | 130.14 (not varied) | 130.14 (not varied) | 130.14 (not varied) |
| First-dose administration,^b^ $ | 27.03 (4.95–27.03); 5.63 | 25.38 (4.95–25.38); 5.21 | 22.56 (4.95–22.56); 4.49 | 8.64 (4.95–8.64); 0.94 | 8.64 (4.95–8.64); 0.94 |
| Second-dose administration,^b^ $ | 32.63 (4.95–32.63); 7.06 | 30.98 (4.95–30.98); 6.64 | 28.16 (4.95–28.16); 5.92 | 14.24 (4.95–14.24); 2.37 | 14.24 (4.95–14.24); 2.37 |
| Vaccine AE costs/dose,^c^ $ | 43.62 (21.81–87.25); 16.69 | 21.05 (10.52–42.10); 8.05 | 20.61 (10.30–41.22); 7.89 | 18.44 (9.22–36.87); 7.05 | 18.44 (9.22–36.87); 7.05 |
| HZ only direct medical costs,^d,e^ $ | 756.67 (454.00–1059.34); 154.42 | 1190.09 (714.06–1666.13); 242.88 | 1190.09 (714.06–1666.13); 242.88 | 1402.84 (841.71–1963.98); 286.29 | 519.54 (311.73–727.36); 106.03 |
| HZ with PHN direct medical costs,^d,e^ $ | 2322.96 (1393.78–3252.14); 474.07 | 3653.57 (2192.14–5114.99); 745.63 | 3653.57 (2192.14–5114.99); 745.63 | 7526.21 (4515.72–10,536.69); 1535.96 | 2787.32 (1672.39–3902.25), 568.84 |
| HZ-related complications,^d,f^ $ | 0 (not varied) | 0 (not varied) | 0 (not varied) | 0 (not varied) | 0 (not varied) |
| Indirect costs per HZ case,^d,g^ $ | 224.41 (179.52–269.29); 22.90 | 224.41 (179.52–269.29); 22.90 | 224.41 (179.52–269.29); 22.90 | 224.41 (179.52–269.29); 22.90 | 224.41 (179.52–269.29); 22.90 |

All costs are in 2022 Canadian dollars

^a^ For the probabilistic base-case analysis, the gamma distribution was assumed for all cost-related inputs. The SEs applied in the gamma distribution parameters are consistent with the reported ranges representing 95% CIs

^b^ Based on vaccine administration costs of $4.95 for the first dose (assumed to be given at a visit for another reason [G538]) or $10.55 for the second dose (assumed to be given at a vaccine-only visit [G700+G538]) [7]. To these, we added costs for 1 hour of work lost for each dose of RZV multiplied by age-specific employment rates from [25] to account for 62% of HSCT recipients returning to work 1 year after transplant [26] and by total compensation per hour worked [8, 9, 27]. The lower bounds were $4.95 for both doses (i.e., vaccination given at a visit for another reason and no indirect cost); the upper bounds were the same as the base-case values

^c^ Based on, for ages 18–49 and ≥ 50 years, respectively, 0.0203 and 0.0170 PCP visits, 0.0045 and 0 ER visits, and 0.0045 and 0.0022 hospitalizations per dose [data on file] at $84.45 (2022 Canadian dollars) per PCP visit due to an allergic reaction [7], $300.48 (2022 Canadian dollars) per ER visit resulting from a reaction to vaccination [28], $7466 (2022 Canadian dollars) per hospitalization (2017–2018 OCCI data for AE-related hospitalizations [ICD-10 T802, T806, T808, T809] [28]) plus work losses of 4 hours per PCP visit, 12 hours per ER visit, and 40 hours per hospitalization multiplied by age-specific employment rates [27] to account for 62% of HSCT recipients returning to work 1 year after transplant [26] and total compensation per hour worked [27]. ER and hospitalization costs were inflated to 2022 Canadian dollars using the health and personal care component of the CPI [8]; wages were inflated to 2022 Canadian dollars using the CPI for all items [8, 9]. Ranges are −50% to +100% of base-case values

^d^ These values apply to individuals with IC status only. Values for individuals with healthy status are shown in Supplemental Table 7

^e^ Direct HZ costs (alone or with PHN) were estimated from Canadian HZ costs from [4-9] multiplied by the ratio of HZ costs for IC versus immunocompetent patients from England [3]. Ranges are ±40%

^f^ We assumed no direct costs specific to non-PHN complications in order to avoid potential double-counting with costs related to HZ with and without PHN from [4]

^g^ Indirect costs were from [24] for HZ alone and HZ with PHN, weighted by the rate of PHN among HZ from [29] and inflated to 2022 using the CPI for all items [8, 9]. Ranges are ±20%

AE, adverse event; CI, confidence interval; CPI, Consumer Price Index; ER, emergency room; HSCT, hematopoietic stem-cell transplant; HZ, herpes zoster; IC, immunocompromised; ICD-10, International Classification of Diseases, 10^th^ revision; OCCI, Ontario Case Costing Initiative; PCP, primary care provider; PHN, postherpetic neuralgia; RZV, recombinant zoster vaccine; SE, standard error

**Supplemental Table 3** Utility inputs for the probabilistic base-case and sensitivity analyses

|  | **Base case (range^a^); SE** | |
| --- | --- | --- |
|  | **Age 18–49 years** | **Age 50–64 years** |
| Baseline utilities^b,c^ [30] | 0.8523 (0.6818–1.0000); 0.0812 | 0.8003 (0.6402–0.9604); 0.0817 |
| QALY loss per HZ only case (unvaccinated)^b,d^ | 0.0144 (0.0106–0.0181); 0.0019 | 0.0144 (0.0106–0.0181); 0.0019 |
| QALY loss per HZ only case (vaccinated)^b,e^ | 0.0047 (0.0034–0.0144); 0.0028 | 0.0047 (0.0034–0.0144); 0.0028 |
| QALY loss per HZ with PHN case (unvaccinated)^b,f^ | 0.1972 (0.0901–0.2879); 0.0505 | 0.1972 (0.0901–0.2879); 0.0505 |
| QALY loss per HZ with PHN case (vaccinated)^b,g^ | 0.1972 (0.1578–0.1972); 0.0101 | 0.1972 (0.1578–0.1972); 0.0101 |
| QALY loss per RZV dose^h^ | 0.000124 (0.000062–0.000248); 0.000047 | 0.000102 (0.000051–0.000205); 0.000039 |

^a^ The beta distribution was assumed for all utility inputs included in the probabilistic base-case analysis. The SEs applied in the beta distribution parameters are consistent with the reported ranges representing 95% CIs

^b^ These values apply to individuals with IC status only. Values for individuals with healthy status are detailed in the “Base-case input parameters” section.

^c^ Ranges are ±20% to a maximum of 1

^d^ Base case (range) was calculated from the mean (95% CI) disutility (assumed ≤ 45 days to symptom relief) from [24] for 45/365 days

^e^ The QALY loss for an unvaccinated HZ case from [24] was adjusted by a 68% reduction in QALY losses for HZ cases in individuals vaccinated with RZV versus unvaccinated HZ cases in [14, 30]. The lower bound is 68% lower than the lower bound for an unvaccinated HZ case; the upper bound is equal to the base-case for an unvaccinated HZ case

^f^ Base case was calculated from the mean disutility (assumed ≤ 45 days to symptom relief) from [24] × 12.9 months [31, 32] × 30/365. Lower and upper bounds used 5.9 months [24] and 18.8 months [33], respectively

^g^ We conservatively assumed that QALY loss for vaccinated cases who develop PHN is the same as QALY loss for unvaccinated cases who develop PHN. The lower bound is −20% of the base-case value; the upper bound is equal to the base-case value

^h^ Calculated based on QALY losses of 0.0001 (local/general, PCP visit, ER visit) or 0.0082 (hospitalization) from [34] (based on data from [35]) at 0.845, 0.0203, 0.0045, and 0.0045, respectively, per RZV dose for ages 18–49 years and 0.8270, 0.0170, 0, and 0.0022, respectively, per RZV dose for ages ≥ 50 years [data on file]. Ranges are −50% to +100%

CI, confidence interval; ER, emergency room; HSCT, hematopoietic stem-cell transplant; HZ, herpes zoster; IC, immunocompromised; PCP, primary care provider; PHN, postherpetic neuralgia; QALY, quality-adjusted life-year; RZV, recombinant zoster vaccine; SE, standard error

**Supplemental Table 4** Annual probabilities of all-cause mortality for people with healthy status

| **Age group** | **Annual probability of death^a^ (2020) [36]** |
| --- | --- |
| 18–49 years | 0.0011^b^ |
| 50–54 years | 0.0031 |
| 55–59 years | 0.0048 |
| 60–64 years | 0.0074 |
| 65–69 years | 0.0111 |
| 70–74 years | 0.0172 |
| 75–79 years | 0.0286 |
| 80–84 years | 0.0496 |
| 85–89 years | 0.0886 |
| ≥ 90 years | 0.1790 |

^a^ Derived from the annual all-cause mortality rates per 1000 population from [36]

^b^ 2022 population estimates from [37] were used to weight the values for 5-year age groups from [36]

**Supplemental Table 5** Epidemiological inputs for initial and recurrent HZ for people with healthy status

|  | **Mean (range); SE** | | | | | |
| --- | --- | --- | --- | --- | --- | --- |
|  | **18–49 years** | **50–59 years** | **60–64 years** | **65–69 years** | **70–79 years** | **≥ 80 years** |
| Annual incidence, per person-year^a^ | 0.00318 (0.00230–0.00333); 0.00026 | 0.00598 (0.00459–0.00603); 0.00037 | 0.00866 (0.00698–0.00990); 0.00074 | 0.00866 (0.00698–0.01156); 0.00117 | 0.01084 (0.00846–0.01131); 0.00073 | 0.01104 (0.00846–0.01244); 0.00102 |
| HZ cases with PHN,^b^ % | 14.6 (7.3–21.9); 3.72 | 14.6 (11.9–18.25); 1.62 | 20.5 (6.9–25.63); 4.78 | 20.5 (6.9–25.63); 4.78 | 33.8 (18.5–42.25); 6.06 | 33.8 (18.5–42.25); 6.06 |
| HZ cases with ocular complications,^c^ % | 3.16 (1.00–4.64); 0.93 | 2.87 (1.00–4.71); 0.95 | 4.23 (1.00–6.57); 1.42 | 4.23 (1.00–6.57); 1.42 | 4.53 (1.00–6.94); 1.51 | 6.91 (1.00–10.08); 2.32 |
| HZ cases with neurological complications,^c^ % | 1.67 (0.59–2.76); 0.55 | 2.23 (0.60–3.86); 0.83 | 3.17 (1.00–5.21); 1.07 | 3.17 (1.00–5.21); 1.07 | 5.92 (1.00–8.65); 1.95 | 4.88 (1.00–7.57); 1.68 |
| HZ cases with cutaneous complications,^c^ % | 1.12 (0.23–2.00); 0.45 | 1.59 (0.21–2.98); 0.71 | 1.06 (0.00–2.25); 0.57 | 1.06 (0.00–2.25); 0.57 | 2.09 (0.44–3.75); 0.84 | 2.85 (0.77–4.92); 1.06 |
| HZ cases with other non-pain complications,^c^ % | 0.74 (0.02–1.47); 0.37 | 1.59 (0.21–2.98); 0.71 | 1.41 (0.04–2.78); 0.70 | 1.41 (0.04–2.78); 0.70 | 2.09 (0.44–3.75); 0.84 | 2.85 (0.77–4.92); 1.06 |

^a^ Mean incidence rates in 2012 from [38]. For ages 18–49 years, incidence estimates from [38] were weighted by 2020 population size estimates from [37]. Ranges were derived from [5, 38-40] as per [21]. Values for initial HZ were also used for recurrent HZ based on [41]

^b^ Mean values are from [42], with 18–49 years assumed to be the same as 50–59 years based on [43]. Lower bounds were estimated from [5, 22, 42] and upper bounds are mean estimate +25%, except for age 18–49 years, where ±50% was used. It was assumed that PHN was as likely after recurrent HZ as after initial HZ

**^c^** Values are from [43]. Lower bounds are the lower of 1% or derived from [43]; upper bounds were derived from [43]

HZ, herpes zoster; SE, standard error; PHN, post-herpetic neuralgia

**Supplemental Table 6** Waning inputs for people with healthy status

|  | **Mean (range); SE** | |
| --- | --- | --- |
|  | **18–69 years** | **≥ 70 years** |
| Annual waning of RZV efficacy (2 doses),^a^ % [44] | 1.5 (0.0–3.4); 0.77 | 2.3 (0.3–4.4); 1.02 |
| Annual waning of RZV efficacy (1 dose),^b^ % [45] | 5.4 (1.0–7.4); 2.24 for years 1–4; | 5.4 (1.0–7.4); 2.24 for years 1–4; |

^a^ The values from [44] for ages 50–69 years were also applied to those aged 18–49 years

^b^ Assumed to be the same as for zoster vaccine live. Ranges were assumed

RZV, recombinant zoster vaccine; SE, standard error

**Supplemental Table 7** Direct HZ cost inputs for people with healthy status

|  | **Mean (range); SE** | | | |
| --- | --- | --- | --- | --- |
|  | **18–49 years** | **50–59 years** | **60–64 years** | **≥ 65 years** |
| Direct costs per HZ alone case (vaccinated or not),^a^ $ | 117.09 (93.67–140.50); 11.95 | 234.17 (187.34–281.01); 23.90 | 234.17 (187.34–281.01); 23.90 | 276.04 (220.83–331.25); 28.17 |
| Direct costs per HZ with PHN case (vaccinated or not),^b^ $ | 359.46 (222.86–496.05); 69.69 | 718.91 (445.73–992.10); 139.38 | 718.91 (445.73–992.10); 139.38 | 1480.93 (918.18–2043.69); 287.12 |
| Indirect costs per HZ case (vaccinated or not),^c^ $ | 550.92 (440.74–661.10); 56.22 | 2203.68 (1762.94–2644.41); 224.86 | 1221.56 (977.25–1465.87); 124.65 | 256.09 (204.87–307.30); 26.13 |

All costs are in 2022 Canadian dollars

^a^ Estimated from drug use and healthcare resource utilization from [4, 5] and unit costs from [6, 7] and OCCI data from 2015–2016 inflated to 2022 Canadian dollars using the health and personal care component of the CPI [9]. Value for age 18–49 years was assumed to be half that for ages 50–59 years. Ranges are ±20%

^b^ Estimated from drug use and healthcare resource utilization from [4, 5] and unit costs from [6, 7] and the OCCI data from 2015–2016 inflated to 2022 Canadian dollars using the health and personal care component of the CPI [9]. Value for age 18–49 years was assumed to be half that for ages 50–59 years. Ranges are ±38%

^c^ Estimated from absenteeism and presenteeism losses (in hours) for HZ cases in [46] multiplied by age-specific employment rates [25] and total compensation per hour worked [27]; inflated to 2022 Canadian dollars using the CPI for all items [9]. Value for age 18–49 years was assumed to be 25% of the cost for ages 50–59 years. Ranges are ±20%

CPI, Consumer Price Index; HZ, herpes zoster; OCCI, Ontario Case Costing Initiative; PHN, post-herpetic neuralgia; SE, standard error

**Supplemental Table 8** Utility inputs for people with healthy status

|  | **Mean (range); SE** | | | | | |
| --- | --- | --- | --- | --- | --- | --- |
|  | **18–49 years** | **50–59 years** | **60–64 years** | **65–69 years** | **70–79 years** | **≥ 80 years** |
| Baseline utilities^a^ | 0.9304 (0.7443–1); 0.0652 | 0.9218 (0.7375–1); 0.067 | 0.9342 (0.7474–1); 0.0644 | 0.9342 (0.7474–1); 0.0644 | 0.9342 (0.7474–1); 0.0644 | 0.868 (0.6944–1); 0.078 |
| QALY losses per HZ only case^b^ | 0.0045 (0.003–0.006); 0.0008 | 0.009 (0.006–0.012); 0.0015 | 0.01 (0.006–0.013); 0.0018 | 0.01 (0.006–0.013); 0.0018 | 0.01 (0.007–0.014); 0.0018 | 0.01 (0.007–0.014); 0.0018 |
| QALY losses per HZ with PHN case^b^ | 0.0205 (0.016–0.026); 0.0026 | 0.041 (0.032–0.052); 0.0051 | 0.192 (0.103–0.29); 0.0477 | 0.192 (0.103–0.29); 0.0477 | 0.234 (0.191–0.29); 0.0253 | 0.234 (0.191–0.29); 0.0253 |

^a^ Estimated based on EQ-5D index values from [47] weighted using 2020 population estimates from [37]. Ranges are ±20% (to a maximum of 1)

^b^ Means and ranges from [48], with those for age 18–49 years assumed to be half those for age 50–59 years

EQ-5D, EuroQol 5 dimensions; HZ, herpes zoster; PHN, postherpetic neuralgia; QALY, quality-adjusted life-year; SE, standard error

**Supplemental Table 9** Direct HZ cost inputs for patients with non-HSCT IC

|  | **Mean (range); SE** | | | |
| --- | --- | --- | --- | --- |
|  | **18–49 years** | **50–64 years** | **65–69 years** | **≥ 70 years** |
| Direct costs per HZ alone case,^a^ $ |  |  |  |  |
| Breast cancer | 231.02 (138.61–323.42); 47.15 | 408.38 (245.03–571.74); 83.34 | 481.39 (288.83–673.95); 98.24 | 412.24 (247.35–577.14); 84.13 |
| Renal transplant | 501.63 (300.98–702.28); 102.37 | 671.77 (403.06–940.48); 137.10 | 791.86 (475.12–1108.61); 161.60 | 375.54 (225.33–525.76); 76.64 |
| HIV | 137.17 (82.30–192.03); 27.99 | 1075.85 (645.51–1506.19); 219.56 | 1268.18 (760.91–1775.45); 258.81 | 276.04 (165.62–386.45); 56.33 |
| Hodgkin lymphoma | 537.84 (322.70–752.97); 109.76 | 823.07 (493.84–1152.30); 167.97 | 970.21 (582.13–1358.29); 198.00 | 542.02 (325.21–758.82); 110.62 |
| Direct costs per HZ with PHN case,^a^ $ |  |  |  |  |
| Breast cancer | 709.22 (425.53–992.90); 144.74 | 1253.73 (752.24–1755.22); 255.86 | 2582.64 (1549.58–3615.69); 527.07 | 2211.67 (1327.00–3096.34); 451.36 |
| Renal transplant | 1539.98 (923.99–2155.97); 314.28 | 2062.33 (1237.40–2887.26); 420.88 | 4248.32 (2548.99–5947.65); 867.00 | 2014.77 (1208.86–2820.67); 411.18 |
| HIV | 421.10 (252.66–589.54); 85.94 | 3302.84 (1981.71–4623.98); 674.05 | 6803.73 (4082.24–9525.22); 1,388.52 | 1480.93 (888.56–2073.30); 302.23 |
| Hodgkin lymphoma | 1651.14 (990.69–2311.60); 336.97 | 2526.81 (1516.09–3537.54); 515.68 | 5205.14 (3123.08–7287.20); 1,062.27 | 2907.90 (1744.74–4071.06); 593.45 |

All costs are in 2022 Canadian dollars

^a^ Estimated based on costs for an immunocompetent population shown in Supplemental Table 7 multiplied by the ratio of costs for each specific IC population versus an immunocompetent population in England [3]. Ranges are ±40%

HIV, human immunodeficiency virus; HSCT, hematopoietic stem-cell transplant; HZ, herpes zoster; IC, immunocompromised; PHN, postherpetic neuralgia; SE, standard error

**Supplemental Fig. 1** Incremental costs versus incremental QALYs from 5000 probabilistic base-case analysis simulations of RZV versus no HZ vaccine for Canadian HSCT adults aged 55 years

CAD, Canadian dollars; HSCT, hematopoietic stem-cell transplant; HZ, herpes zoster; QALY, quality-adjusted life-year; RZV, recombinant zoster vaccine

**Supplemental Fig. 2** ICERs for RZV versus no HZ vaccine for select populations of Canadian IC adults aged (a) 45 years, (b) 35 years, and (c) 25 years at model start, across ranges of values for the IC status duration and the annual HZ incidence


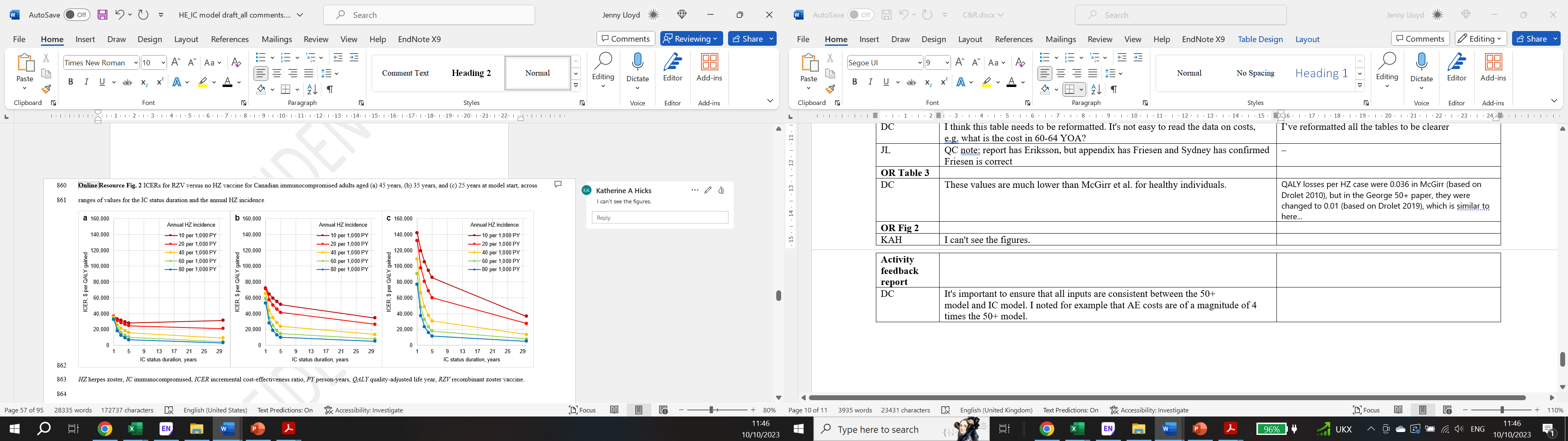


HZ, herpes zoster; IC, immunocompromised; ICER, incremental cost-effectiveness ratio; PY, person-years; QALY, quality-adjusted life year; RZV, recombinant zoster vaccine

**Supplemental references**

1. Su SH, Martel-Laferriere V, Labbe AC, Snydman DR, Kent D, Laverdiere M, et al. High incidence of herpes zoster in nonmyeloablative hematopoietic stem cell transplantation. Biol Blood Marrow Transplant. 2011;17(7):1012-7.

2. Sahoo F, Hill JA, Xie H, Leisenring W, Yi J, Goyal S, et al. Herpes zoster in autologous hematopoietic cell transplant recipients in the era of acyclovir or valacyclovir prophylaxis and novel treatment and maintenance therapies. Biol Blood Marrow Transplant. 2017;23(3):505-11.

3. Curran D, Hunjan M, El Ghachi A, El-Hahi Y, Bianco V, Ferreira G. Herpes zoster related healthcare burden and costs in immunocompromised (IC) and IC-free populations in England: An observational retrospective database analysis. BMJ Open. 2019;9(8):e023502.

4. Friesen KJ, Chateau D, Falk J, Alessi-Severini S, Bugden S. Cost of shingles: Population based burden of disease analysis of herpes zoster and postherpetic neuralgia. BMC Infect Dis. 2017;17(1):69.

5. Najafzadeh M, Marra CA, Galanis E, Patrick DM. Cost effectiveness of herpes zoster vaccine in Canada. Pharmacoeconomics. 2009;27(12):991-1004.

6. Ontario Ministry of Health and Long-Term Care. Formulary search. <https://www.formulary.health.gov.on.ca/formulary/>. Accessed

7. Ontario Ministry of Health. Schedule of benefits. Physician services under the Health Insurance Act. <https://www.health.gov.on.ca/en/pro/programs/ohip/sob/physserv/sob_master_20221201.pdf>. Accessed 31 Mar 2022.

8. Statistics Canada. Consumer Price Index, annual average, not seasonally adjusted. <https://doi.org/10.25318/1810000501-eng>. Accessed 31 Mar 2022.

9. Statistics Canada. Consumer Price Index, monthly, not seasonally adjusted. <https://doi.org/10.25318/1810000401-eng>. Accessed 1 Apr 2022.

10. Organisation for Economic Co-operation and Development. <https://stats.oecd.org/index.aspx?DataSetCode=SHA>. Accessed Aug 8, 2022.

11. Lal H, Cunningham AL, Godeaux O, Chlibek R, Diez-Domingo J, Hwang SJ, et al. Efficacy of an adjuvanted herpes zoster subunit vaccine in older adults. N Engl J Med. 2015;372(22):2087-96.

12. Cunningham AL, Lal H, Kovac M, Chlibek R, Hwang SJ, Diez-Domingo J, et al. Efficacy of the herpes zoster subunit vaccine in adults 70 years of age or older. N Engl J Med. 2016;375(11):1019-32.

13. Dagnew AF, Ilhan O, Lee WS, Woszczyk D, Kwak JY, Bowcock S, et al. Immunogenicity and safety of the adjuvanted recombinant zoster vaccine in adults with haematological malignancies: A phase 3, randomised, clinical trial and post-hoc efficacy analysis. Lancet Infect Dis. 2019;19(9):988-1000.

14. Bastidas A, de la Serna J, El Idrissi M, Oostvogels L, Quittet P, Lopez-Jimenez J, et al. Effect of recombinant zoster vaccine on incidence of herpes zoster after autologous stem cell transplantation: A randomized clinical trial. JAMA. 2019;322(2):123-33.

15. Blank LJ, Polydefkis MJ, Moore RD, Gebo KA. Herpes zoster among persons living with HIV in the current antiretroviral therapy era. J Acquir Immune Defic Syndr. 2012;61(2):203-7.

16. Pergam SA, Forsberg CW, Boeckh MJ, Maynard C, Limaye AP, Wald A, et al. Herpes zoster incidence in a multicenter cohort of solid organ transplant recipients. Transpl Infect Dis. 2011;13(1):15-23.

17. Habel LA, Ray GT, Silverberg MJ, Horberg MA, Yawn BP, Castillo AL, et al. The epidemiology of herpes zoster in patients with newly diagnosed cancer. Cancer Epidemiol Biomarkers Prev. 2013;22(1):82-90.

18. McKay SL, Guo A, Pergam SA, Dooling K. Herpes zoster risk in immunocompromised adults in the United States: A systematic review. Clin Infect Dis. 2020;71(7):e125-e34.

19. Chen SY, Suaya JA, Li Q, Galindo CM, Misurski D, Burstin S, et al. Incidence of herpes zoster in patients with altered immune function. Infection. 2014;42(2):325-34.

20. Brisson M. Estimating the number needed to vaccinate to prevent herpes zoster-related disease, health care resource use and mortality. Can J Public Health. 2008;99(5):383-6.

21. McGirr A, Van Oorschot D, Widenmaier R, Stokes M, Ganz ML, Jung H, et al. Public health impact and cost-effectiveness of non-live adjuvanted recombinant zoster vaccine in Canadian adults. Appl Health Econ Health Policy. 2019;17(5):723-32.

22. Brisson M, Pellissier JM, Camden S, Quach C, De Wals P. The potential cost-effectiveness of vaccination against herpes zoster and post-herpetic neuralgia. Hum Vaccin. 2008;4(3):238-45.

23. Edgar BL, Galanis E, Kay C, Skowronski D, Naus M, Patrick D. The burden of varicella and zoster in British Columbia 1994-2003: Baseline assessment prior to universal vaccination. Can Commun Dis Rep. 2007;33(11):1-15.

24. Eriksson J, Hunger M, Bourhis F, Thoren R, Popmihajlov Z, Finelli L, et al. Cost and utility in immunocompromised subjects who developed herpes zoster during the randomized V212 inactivated varicella-zoster vaccine (ZV_IN_) trial. Expert Rev Pharmacoecon Outcomes Res. 2020;20(6):613-21.

25. Statistics Canada. Labour force characteristics, monthly, seasonally adjusted and trend-cycle, last 5 months. <https://doi.org/10.25318/1410028701-eng>. Accessed 5 Apr 2022.

26. Morrison EJ, Ehlers SL, Bronars CA, Patten CA, Brockman TA, Cerhan JR, et al. Employment status as an indicator of recovery and function one year after hematopoietic stem cell transplantation. Biol Blood Marrow Transplant. 2016;22(9):1690-5.

27. Statistics Canada. Labour productivity and related measures by business sector industry and by non-commercial activity consistent with the industry accounts. <https://doi.org/10.25318/3610048001-eng>. Accessed 5 Apr 2022.

28. Ontario Ministry of Health and Long-Term Care. Ontario Case Costing Initiative Costing Analysis Tool. Acute inpatient FY 2017/2018 statistics by most responsible diagnosis ICD-10 Code. <https://hsim.health.gov.on.ca/hdbportal/>. Accessed 15 Jun 2021.

29. Winston DJ, Mullane KM, Cornely OA, Boeckh MJ, Brown JW, Pergam SA, et al. Inactivated varicella zoster vaccine in autologous haemopoietic stem-cell transplant recipients: An international, multicentre, randomised, double-blind, placebo-controlled trial. Lancet. 2018;391(10135):2116-27.

30. Curran D, Matthews S, Rowley SD, Young JH, Bastidas A, Anagnostopoulos A, et al. Recombinant zoster vaccine significantly reduces the impact on quality of life caused by herpes zoster in adult autologous hematopoietic stem cell transplant recipients: A randomized placebo-controlled trial (ZOE-HSCT). Biol Blood Marrow Transplant. 2019;25(12):2474-81.

31. Moore L, Remy V, Martin M, Beillat M, McGuire A. A health economic model for evaluating a vaccine for the prevention of herpes zoster and post-herpetic neuralgia in the UK. Cost Eff Resour Alloc. 2010;87.

32. Oxman MN, Levin MJ, Johnson GR, Schmader KE, Straus SE, Gelb LD, et al. A vaccine to prevent herpes zoster and postherpetic neuralgia in older adults. N Engl J Med. 2005;352(22):2271-84.

33. Lieu TA, Ortega-Sanchez I, Ray GT, Rusinak D, Yih WK, Choo PW, et al. Community and patient values for preventing herpes zoster. Pharmacoeconomics. 2008;26(3):235-49.

34. Le P, Rothberg MB. Cost-effectiveness of herpes zoster vaccine for persons aged 50 years. Ann Intern Med. 2015;163(7):489-97.

35. Schmader KE, Levin MJ, Gnann JW, Jr., McNeil SA, Vesikari T, Betts RF, et al. Efficacy, safety, and tolerability of herpes zoster vaccine in persons aged 50-59 years. Clin Infect Dis. 2012;54(7):922-8.

36. Statistics Canada. Mortality rates, by age group. <https://doi.org/10.25318/1310071001-eng>. Accessed 31 Mar 2022.

37. Statistics Canada. Population estimates on July 1st, by age and sex. <https://doi.org/10.25318/1710000501-eng>. Accessed 31 Mar 2022.

38. Marra F, Chong M, Najafzadeh M. Increasing incidence associated with herpes zoster infection in British Columbia, Canada. BMC Infect Dis. 2016;16(1):589.

39. Russell ML, Dover DC, Simmonds KA, Svenson LW. Shingles in Alberta: Before and after publicly funded varicella vaccination. Vaccine. 2014;32(47):6319-24.

40. Tanuseputro P, Zagorski B, Chan KJ, Kwong JC. Population-based incidence of herpes zoster after introduction of a publicly funded varicella vaccination program. Vaccine. 2011;29(47):8580-4.

41. Yawn BP, Wollan PC, Kurland MJ, St Sauver JL, Saddier P. Herpes zoster recurrences more frequent than previously reported. Mayo Clin Proc. 2011;86(2):88-93.

42. Drolet M, Brisson M, Schmader K, Levin M, Johnson R, Oxman M, et al. Predictors of postherpetic neuralgia among patients with herpes zoster: A prospective study. J Pain. 2010;11(11):1211-21.

43. Yawn BP, Saddier P, Wollan PC, St Sauver JL, Kurland MJ, Sy LS. A population-based study of the incidence and complication rates of herpes zoster before zoster vaccine introduction. Mayo Clin Proc. 2007;82(11):1341-9.

44. Curran D, Van Oorschot D, Matthews S, Hain J, Salem AE, Schwarz M. Long-term efficacy data for the recombinant zoster vaccine: Impact on public health and cost effectiveness in Germany. Hum Vaccin Immunother. 2021;17(12):5296-303.

45. Curran D, Van Oorschot D, Varghese L, Oostvogels L, Mrkvan T, Colindres R, et al. Assessment of the potential public health impact of herpes zoster vaccination in Germany. Hum Vaccin Immunother. 2017;13(10):2213-21.

46. Drolet M, Levin MJ, Schmader KE, Johnson R, Oxman MN, Patrick D, et al. Employment related productivity loss associated with herpes zoster and postherpetic neuralgia: A 6-month prospective study. Vaccine. 2012;30(12):2047-50.

47. Szende A, Janssen B, Cabases J. Self-reported population health: An international perspective based on EQ-5D. Dordrecht, The Netherlands: Springer Netherlands; 2014.

48. Drolet M, Zhou Z, Sauvageau C, DeWals P, Gilca V, Amini R, et al. Effectiveness and cost-effectiveness of vaccination against herpes zoster in Canada: A modelling study. CMAJ. 2019;191(34):E932-9.
